# The Financial Burden Associated with Medical Costs among Childhood Cancer Patients and Their Families Related to Their Socioeconomic Status: The Perspective of National Health Insurance Service

**DOI:** 10.3390/ijerph17176020

**Published:** 2020-08-19

**Authors:** Wonjeong Chae, Juyeong Kim, Sohee Park, Eun-Cheol Park, Sung-In Jang

**Affiliations:** 1 Department of Public Health, College of Medicine, Yonsei University, Seoul 03722, Korea; wjchae0816@yuhs.ac; 2 Institute of Health Services Research, Yonsei University, Seoul 03722, Korea; ecpark@yuhs.ac; 3 Department of Health & Human Performance, Sahmyook University, Seoul 01795, Korea; kjy394@syu.ac.kr; 4 Department of Biostatistics, Graduate School, Yonsei University, Seoul 03722, Korea; soheepark@yuhs.ac; 5 Department of Preventive Medicine, College of Medicine, Yonsei University, Seoul 03722, Korea

**Keywords:** childhood cancer, medical cost, socioeconomic status, financial burden, national health insurance, Korea

## Abstract

The number of cancer survivors is increasing as a consequence of improved therapeutic options. Many families are suffering from the resultant financial burden. Our study aims to determine the total medical cost for 5 years after the initial diagnosis of childhood cancers. A customized dataset from the Korean National Health Insurance Claims Database was requested for this study. A total of 7317 patients were selected to determine the total medical cost. The costs are presented as the 2% trimmed mean value to exclude extreme costs. The medical costs were further classified according to cancer type, treatment phase, and socioeconomic status. Multiple linear regression analyses were performed. The average total medical cost per patient is 36.8 million Korean Wons or 32,157 United States Dollars. Analysis of socioeconomic status revealed that the higher income group demonstrated higher medical expenditure when compared to other groups. Analysis of the treatment phase showed that costs associated with the early phase of treatment are the highest, especially in the first 3 months after initial diagnosis. To alleviate the financial burden and reduce the socioeconomic disparities associated with medical care and costs, a better understanding of the current experience of patients and their families is required.

## 1. Introduction

Cancer is the second leading cause of death globally. According to a World Health Organization report, cancer resulted in 9.6 million deaths in 2018 [1]. In addition, cancers are a leading cause of death in children and adolescents and their prevalence has been increasing since globally [2,3,4,5]. In high-income countries, cancer is the number one cause of disease-related death [2,6]. 

Although deaths from childhood cancers are still high, childhood cancer is curable with advanced medical therapy and technology. Several years of research have improved treatment options for cancer patients, thus improving their survival rate [7,8]. In the United States, the 5-year survival rate for childhood cancer in 2020 is 84%, whereas it was 58% in the mid-1970s and 79% in the mid-1990s [9]. In the United Kingdom, the 5-year survival rate in 2020 is between 76% and 82%, whereas it was 36% in 1970 [10]. Also, South Korea (Korea) shows improvement in the 5-year survival rate improving from 52% in the 1980s to over 80% by 2020 [11,12,13].

Along with the increased number of childhood cancer cases and survivors, the cost of these treatments and the financial burden on patients and their families are high [14,15,16,17,18]. The cost of childhood cancer treatment is also high in the United States and begins at 833,000 United States Dollars (USD) [16]. The medical expense associated with childhood cancer treatment and care has also increased to 87.7 billion Korean Wons (KRW) in 2014 from KRW 83.1 billion in 2010 [5]. 

For childhood cancer patients, the financial burden on their family is higher than that associated with treatment for adult cancer patients [17,19,20,21]. A study by Merrill et al. reported that the average cost per day for childhood cancer treatments was 700 USD higher than that of adult cancer treatments [22]. Additionally, the hospitalization cost for leukemia patients was higher in children at 55,700 USD compared with adults at 40,200 USD [23]. 

Families of childhood cancer patients often experience disruptions in the regular employment of the caregiver, which significantly diminishes the household income [24,25,26]. Some studies show that families with childhood cancer patients are driven below the poverty line due to high medical costs [24,26,27,28]. Furthermore, some patients and their families make treatment decisions based on the associated cost, which can lead to undesirable outcomes [28,29]. Therefore, it is important to prioritize and allocate the limited healthcare resources in an efficient manner and provide financial support to patients and their families. 

Understanding the economic impact of childhood cancer is essential. There are very few studies describing the financial burden associated with medical treatments and its impact on patients’ families [22,24]. The purpose of this study is to investigate the burden of childhood cancer costs on cancer survivors and their families based on socioeconomic status. To this end, we examined the medical costs associated with cancer survival for 5 years after the initial cancer diagnosis, based on childhood cancer type and socioeconomic status.

## 2. Materials and Methods 

### 2.1. Data 

The National Health Insurance Service (NHIS) database includes medical diagnoses, treatments, drug information, hospital information, and demographic information. Medical diagnoses are indicated using International Statistical Classification of Diseases and Related Health Problems, 10th revision (ICD-10) codes. For this study, we requested a customized dataset of children who were diagnosed with cancer (C00-C99) and their parents’ income data from 2002 to 2015 from the Korean National Health Insurance Claims Database.

### 2.2. National Health Insurance in Korea

Korea has a Universal Health Insurance system that has a single insurer, NHIS. The compulsory premium is deducted based on the individual’s income. However, there is no difference in receiving healthcare services. National Health Insurance (NHI) has two sections, which are covered services and non-covered services. The NHI has 65% of covered medical service items and 35% of non-covered items for medical services. Those items in non-covered services are usually non-essential services and expensive items. 

In general, the out-of-pocket (OOP) money for inpatient care services in covered items is 20%, while outpatient care services in the covered items are 30–50%. The OOP for non-covered items services is 100%. From the day of diagnosis, the cancer patient’s OOP for inpatient care and outpatient care services reduces to 5% for 5 years. Therefore, in this study, OOP for patients was 5% of the medical cost. 

### 2.3. Study Population

From a total of 21,205 patients aged 0–17 who were diagnosed with cancer, we applied a 1-year washout period to identify and include newly diagnosed cancer patients for the study. To obtain socioeconomic status, we extrapolated insurance payment data from the patient’s parent income data and linked the two parameters together. We excluded 5598 patients who lacked parent data. Our study design also excluded cancer patients that died within 5 years of diagnosis. Finally, we excluded 150 patients whose medical expenditure was in the upper and lower 1% of all medical costs. A total of 7317 patients were ultimately used for the study.

### 2.4. Medical Cost

Medical costs in our study were obtained from NHI claim data, which are the real costs of medical services. Thus, we are presenting the insurer’s perspective as average costs (mean values). To eliminate the extreme values, we applied a 2% trimmed mean and excluded the upper and lower 1% values. The cost includes all medical treatment expenses for 5 years after the initial diagnosis. For costs between the years 2003 to 2014, we applied an adjustment in the unit price per score to reflect the actual value of the cost in the year 2015. The medical costs were corrected by multiplying the unit price by the following adjustment rates: 1.30 (2003), 1.26 (2004), 1.23 (2005), 1.19 (2006), 1.15 (2007), 1.13 (2008), 1.10 (2009), 1.09 (2010), 1.08 (2011), 1.06 (2012), 1.04 (2013), and 1.02 (2014). In this study, all amounts are presented in KRW [1 USD is approximately equivalent to 1144.40 KRW on average for the year 2015]. 

### 2.5. Cancer Types 

In our study, we grouped childhood cancers into six categories based on the International Classification of Childhood Cancer [30] and Korean Central Cancer Registry Annual Report [31]: leukemia (C91-C95), brain and central nervous system cancer (C70-C72), non-Hodgkin’s lymphoma (C82-C85, C96), bone and articular cartilage cancer (C40-C41), mesothelioma soft tissues (C45-C49), and the remaining group of ‘other’ cancers. We classified cancer treatment into three phases: early, middle, and late phases. The early phase is the first year following diagnosis, the middle phase includes the second to the fourth year following diagnosis, and the late phase is the fifth year following diagnosis. The early phase has been further divided into 3, 6, 9, and 12 months.

### 2.6. Socioeconomic Status

Socioeconomic status is classified based on the parents’ health insurance premium, which is reflective of the household income. The status is categorized into the following quartile ranges: low (KRW 0−43,260,000), mid-low (KRW 43,261,000−74,555,000), mid-high (KRW 75,556,000−122,370,000), and high (KRW 122,371,000 or more). 

### 2.7. Statistical Analysis

The main analysis was performed using multivariable linear regression. The analysis was performed to determine the association between the outcome variable, cost of cancer treatments, and covariates. For sub-group analysis, we performed multivariable linear regressions based on the patients’ socioeconomic status. All analyses were performed using the SAS software, version 9.4 (SAS Institute, Cary, NC, USA).

## 3. Results

### 3.1. General Characteristics of Study Population

Table 1 shows the general characteristics of the study population. In our study, we included 7317 patients, of whom 27.8% were leukemia patients, 15.2% were brain and central nervous system cancer patients, 9.2% were non-Hodgkin’s lymphoma patients, 7.0% were bone and articular cartilage cancer patients, 5.1% were mesothelioma soft tissue cancer patients, and 35.8% were ‘other’ cancer patients. 

### 3.2. Average Medical Cost of Childhood Cancer Survivors for 5 Years from the Diagnosis

Table 2 displays the average medical cost incurred by childhood cancer survivors from the time of initial diagnosis until the end of the next 5 years. The total medical cost per patient is 36.8 million KRW (Approx. USD 32,157). Inpatient services cost 28.3 million KRW (Approx. USD 24,729), whereas outpatient services cost 8 million KRW (Approx. USD 6991). When classified by cancer type, leukemia was associated with the highest cost at 53.5 million KRW (Approx. USD 46,749) followed by bone and articular cartilage cancer at 52.0 million KRW (Approx. USD 45,439) (Supplementary Table S1).

### 3.3. Regression Analysis on Childhood Cancer Survivors’ Medical Cost and Usage

Results of a regression analysis on total medical costs for childhood cancer survivors are presented in Table 3. Relative to patients in high household income, low household income (β: 5,503,525, *p*-value: <0.001), mid-low household income (β: 4,358,768, *p*-value: <0.001), and mid-high household income (β: 2,099,218, *p*-value: 0.050) groups had less. Patients undergoing chemotherapy, radiotherapy, and surgery bore higher medical costs compared with those who did not receive those major treatments (chemotherapy: β: 22,343,241, *p*-value: <0.001; radiotherapy: β: 17,198,259, *p*-value: <0.001; surgery: β: 19,107,892, *p*-value: <0.001). Patients who went to hospitals in the capital region (β: 1,224,772, *p*-value: 0.128) and metropolitan area (β: 1,332,197, *p*-value: 0.269) cost higher compared to rural area hospitals, yet the difference is not statistically significant. When analyzed according to socioeconomic status, the medical cost was greater in patients of higher socioeconomic status. This result was unaffected by cancer type (Supplementary Table S2). 

To investigate the duration of services received, regression analyses on the number of days of medical service use was performed (Table 4). Leukemia patients used medical services for a longer duration (104.0 days) compared to any other childhood cancer patients in both inpatient (67.4 days) and outpatient (36.6 days) service categories.

### 3.4. Cumulative Medical Cost for 5 Years by Socioeconomic Status

**Figure 1:**
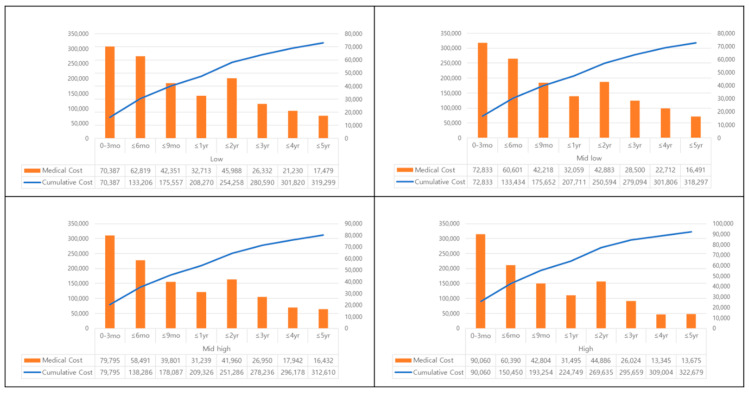
Medical costs of childhood cancer survivor by socioeconomic status in each treatment phase cost and cumulative cost ^†^. † Presented in KRW (Approx. KRW 1140.40 = USD 1 on average, year 2015).

Figure 1 demonstrates the medical costs borne by childhood cancer survivors, based on their socioeconomic status, in each treatment phase and the cumulative medical cost. Medical costs for patients were high in the early phase of treatment. When analyzed according to socioeconomic status, increased medical costs were observed in the first 3 months following diagnosis for the high status category. The medical costs based on socioeconomic status are as follows: low household income group at 11.7 million KRW (USD 9776); mid-low at 12.1 million KRW (USD 10,116); mid-high at 12.2 million KRW (USD 11,083); and high at 15.0 million KRW (USD 12,508) for the first 3 months of the treatment phase (Supplementary Table S3). 

## 4. Discussion

Our study focused on the medical treatment-associated financial burden borne by childhood cancer patients and their families based on their socioeconomic status. Childhood cancer impacts not only the patient, but also the family [17,18,21,32]. In our study, we found that medical expenditure varied based on socioeconomic status and treatment phase in Korea. Previous studies on the financial burden of childhood cancers were limited to specific hospitals or areas with small sample sizes. Because our study utilized NHIS data, which is the real data of the medical cost, our sample size is greater and more representative of the Korean population compared to previous studies. 

We found that the medical expenditure at early phases of treatment is the highest. Clinical research has reported that during the early phases of cancer treatment, intensive care, lengthy hospitalization, and frequent outpatient services are needed. Therefore, after cancer diagnosis, patients and their families experience financial stress in addition to the psychological stress associated with the diagnosis itself [14,17,25,27]. Similar to our results, Warner et al. reported that in the first year of diagnosis, childhood cancer patients and families are financially vulnerable [28]. Considering that the main caregiver for the patient is a parent, the inevitable disruption of parents’ employment in many cases increases the financial burden on the family [15,21,27,32]. Although this financial burden affects families independently of socioeconomic status, patients and their families in the low status category suffer more [27,28,33,34,35,36].

Cancer treatment requires long-term care that can place an immense financial burden on patients’ families [37,38]. Some patients could make treatment decisions based on costs [19,34,35,36,39]. To reduce this financial burden, governments and organizations have developed programs to aid patient’s and family’s financial burden. For example, the Korean government has a special system in place wherein a cancer patient pays only 5% of the total OOP costs covered for 5 years after the diagnosis [37,40]. The United States has government support care programs, local support networks, and pharmaceutical assistant programs to financially assist financially [41]. Also, the United Kingdom have government grants and charity grants for cancer patients [42]. While this system provides some relief to patients and families, the treatments are expensive, and some facets of treatment are not covered by the national health insurance. As our results demonstrate, the medical cost for childhood cancer survivors varies based on socioeconomic status. Importantly, the results only include treatments covered by insurance. In addition to the special system described above, the government also provides financial support to low-income patients who pay health insurance premiums below KRW 100,000 [40]. However, our study found that the medical costs of the low status group and the mid-low status group showed not much of a difference on average. The mid-low status group had decreased cumulative medical costs at 1-year post-diagnosis. Patients did not have different medical accessibility or service based on their socioeconomic status. However, due to the uncovered items and 20% of OOP cost, patients could have received different services. Thus, it could cause difference in medical services. Currently, most supportive programs target patients in low socioeconomic status based on the household income. Yet, people in the mid-low group have limited support programs towards medical treatment. Therefore, we suggest expanding financial support for families who are in need.

Studies on the financial burden of cancer patients were conducted for years, yet few studies on the financial burden of childhood cancer patients and their families were conducted. Childhood cancer patients require long term follow-up care and support to adjust in society after they spent most of their time at the hospital [37,38,43,44]. They have higher risks of developing second cancer and complications [44,45,46,47]. All those factors led to expensive medical costs [14,24,28,29,38]. Furthermore, when cancer is diagnosed, patients start treatment so that they can avoid metastasis and obtain successful results from the treatment [48,49,50]. Also, intensive treatments in the early treatment phase can reduce the recurrence of cancer [48]. Thus, the patient and family often want to start advanced treatment immediately, despite the higher cost. Often those expensive treatments are ones that the patient and family suffer from financial burden [50,51,52]. The overall treatment plan could be affected due to financial reasons [52,53]. Unfortunately, patients with low socioeconomic status are those who tend to alter the treatment plan [14,16,33,53]. 

Although our study population was under a universal health insurance system, there was a difference among socioeconomic statuses. We believe the reason behind the difference in medical costs among the socioeconomic status is on the costs of uncovered items. If we factored in non-covered treatments in our calculations, it would further exacerbate the gap in financial burden based on socioeconomic status. Cancer treatments are using advanced technology. There are treatments that private insurance would not cover. Thus, patients face high OOP costs, and difficulty in access to medical treatments varies depending on their financial capability [19,35,54,55]. For pediatric cancer patients, long term care in hospitals, and frequent outpatient visits are needed [56,57] that the difference in doctor’s visit due to socioeconomic level could lead to different outcomes.

The socioeconomic status should not determine the end outcome of the disease. The high medical costs associated with cancer treatments, governments, and various organizations have established a financial support program for patients. However, the financial burden on families continues to remain high [14,15,16,17,19,20,28,32,58]. Under the national health insurance system in Korea, the essential treatment will be given equally to patients regardless of premium. Even with NHI and its covered items, which are essential services compared to non-covered items, there are differences. The difference lies in whether patients will use services at the minimum level or use services at an insufficient level. The use of the intensive care unit is an example. There may be a difference between using the intensive care unit after surgery for a sufficient period of time with an emphasis on the safety of the patient or using the intensive care unit for a minimum period of time. The latter would place an emphasis on the cost of the care, and the patient would be more quickly transferred to the general ward. We suggest installing the proper allocation of medical resources and institutional support systems in order to lower the associated financial burden on the patients and their families [14,19,20,28]. 

There are some limitations to our study. First, the medical expenses calculated in our study only indicate those covered by health insurance. We were unable to obtain non-covered medical expenses; most costs related to childhood cancers are covered by national health insurance based on the Universal Health Insurance system in Korea. Secondly, we did not include the medical expenses associated with subsequent non-cancer-related to doctor visits and treatments. As cancer treatments comprise very intensive care, we expect that other treatment costs would not substantially alter our results. Thirdly, we only considered whether the patients received treatments such as surgery, chemotherapy, and radiation, but not the frequency at which they received them. Also, we were not able to distinguish the stage of cancer and the specific treatment for each cancer type. Fourthly, the number of cancer cases in our study is less than the actual societal rate. For the study, we included patients who have their patients’ data so that we can measure their socioeconomic status. Lastly, the medical cost calculated for our study includes the direct cost only. We were not able to obtain indirect cost from the insurance claim data. Therefore, further studies are necessary to overcome these limitations. 

## 5. Conclusions

In conclusion, as improved medical treatments increase the numbers of childhood cancer survivors, the economic burden associated with medical treatment also increases. A better understanding of how the financial burden is unevenly distributed across socioeconomic classes is required to reduce the disparities in medical treatment received by patients. In addition, considering the duration of healthcare needed for patients, establishing a continuous economic support policy would be beneficial for lowering the financial burden on childhood cancer patients and their families.

## Figures and Tables

**Table 1:** General characteristics of the study population.

Variable	5-Year Cancer Survivors	
	*N*	%
	7317	100.0
**Sex**		
Male	3978	54.4
Female	3339	45.6
**Age at diagnosis**		
0–4	2220	30.3
5–9	1545	21.1
10–14	2048	28.0
15–17	1504	20.6
**Household income level**		
Low	2223	30.4
Mid-low	2118	29.0
Mid-high	1759	24.0
High	1217	16.6
**Treated hospital type**		
Tertiary hospital	7258	99.2
Other	59	0.8
**Hospital region**		
Capital Area	4656	63.6
Metropolitan Area	820	11.2
Rural Area	1841	25.2
**Chemotherapy**		
Yes	4902	67.0
No	2415	33.0
**Radiotherapy**		
Yes	1891	25.8
No	5426	74.2
**Surgery**		
Yes	3909	53.4
No	3408	46.6
**Cancer type**		
Leukemia	2031	27.8
Brain and central nervous system cancer	1111	15.2
Non-Hodgkin lymphoma	672	9.2
Bone and articular cartilage cancer	511	7.0
Soft tissues	372	5.1
Other cancers	2620	35.8

**Table 2:** Total medical cost of childhood cancer survivors from the initial diagnosis (mean ± S.D.).

Variable	Total Medical Cost ^†^		
	Mean	±	S.D
**Total**	**36,799**	**±**	**37,485**
**Sex**			
Male	39,350	±	38,670
Female	33,795	±	36,073
**Age at diagnosis**			
0–4	36,583	±	35,594
5–9	40,334	±	38,287
10–14	39,123	±	39,301
15–17	30,399	±	36,645
**Household income level**			
Low	33,081	±	36,202
Mid-low	35,701	±	36,582
Mid-high	38,996	±	38,479
High	42,421	±	39,720
**Treated hospital type**			
Tertiary hospital	37,071	±	37,642
Other	5354	±	9388
**Hospital region**			
Capital Area	39,412	±	37,858
Metropolitan Area	31,780	±	37,755
Rural Area	32,490	±	36,273
**Chemotherapy**			
Yes	50,805	±	37,890
No	8418	±	12,934
**Radiotherapy**			
Yes	52,635	±	42,948
No	31,301	±	33,867
**Surgery**			
Yes	41,309	±	42,003
No	31,660	±	31,041
**Medical service**			
Inpatient	28,321	±	23,717
Outpatient	8494	±	7180

† Presented in KRW (Approx. KRW 1140.40 = USD 1.00, on average year 2015). Unit: 1000 KRW.

**Table 3:** Result of regression analysis on the total medical costs to childhood cancer survivors.

Variable	Total Medical Cost ^†^		
	β	S.E	*p*-Value
**Sex**			
Male	2,279,363	679,966	0.001
Female	Ref.		
**Age at diagnosis**			
0–4	3,220,951	1,014,015	0.002
5–9	2,073,293	1,075,761	0.054
10–14	3,566,651	984,064	<0.001
15–17	Ref.		
**Household income level**			
Low	−5,503,525	1,036,325	<0.001
Mid-low	−4,358,768	1,041,386	<0.001
Mid-high	−2,099,218	1,069,302	0.050
High	Ref.		
**Treated hospital type**			
Tertiary hospital	−4,952,756	3,788,808	0.191
Other	Ref.		
**Hospital region**			
Capital Area	1,224,772	805,016	0.128
Metropolitan Area	1,332,197	1,204,805	0.269
Rural Area	Ref.		
**Chemotherapy**			
Yes	33,343,241	812,130	<0.001
No	Ref.		
**Radiotherapy**			
Yes	17,198,259	827,924	<0.001
No	Ref.		
**Surgery**			
Yes	19,107,892	791,327	<0.001
No	Ref.		
**Cancer type**			
Leukemia	29,550,083	1,025,331	<0.001
Brain and central nervous system cancer	11,842,109	1,063,896	<0.001
Non-Hodgkin lymphoma	11,803,352	1,304,556	<0.001
Bone and articular cartilage cancer	23,050,088	1,441,362	<0.001
Mesothelioma Soft tissues	7,583,751	1,589,286	<0.001
Other cancers	Ref.		

† Presented in KRW (Approx. KRW 1140.40 = USD 1.00, on average year 2015).

**Table 4:** Result of regression analysis on the duration of medical service (days).

Variable	Total			Inpatient Service			Outpatient Service		
	β	S.E	*p*-Value	β	S.E	*p*-Value	β	S.E	*p*-Value
**Sex**									
Male	5.8	3.0	0.054	4.6	2.1	0.027	1.2	1.7	0.494
Female	Ref.			Ref.			Ref.		
**Age at diagnosis**									
0–4	51.4	4.5	<0.001	26.8	3.1	<0.001	24.6	2.6	<0.001
5–9	34.5	4.7	<0.001	14.2	3.3	<0.001	20.2	2.8	<0.001
10–14	25.6	4.3	<0.001	13.3	3.0	<0.001	12.3	2.5	<0.001
15–17	Ref.			Ref.			Ref.		
**Household income level**									
Low	−22.7	4.6	<0.001	−4.7	3.1	0.131	−17.9	2.7	<0.001
Mid-low	−17.0	4.6	<0.001	−3.2	3.2	0.303	−13.7	2.7	<0.001
Mid-high	−6.4	4.7	0.173	−1.6	3.2	0.623	−4.8	2.7	0.081
High	Ref.			Ref.			Ref.		
**Treated hospital type**									
Tertiary hospital	−11.0	16.6	0.507	1.7	11.5	0.881	−12.8	9.7	0.190
Other	ref.			ref.			ref.		
**Hospital region**									
Capital Area	−8.2	3.5	0.021	−27.3	2.4	<0.001	19.1	2.1	<0.001
Metropolitan Area	−0.1	5.3	0.989	−5.0	3.6	0.173	4.9	3.1	0.114
Rural Area	Ref.			Ref.			Ref.		
**Chemotherapy**									
Yes	164.0	3.6	<0.001	91.9	2.5	<0.001	72.0	2.1	<0.001
No	Ref.			Ref.			Ref.		
**Radiotherapy**									
Yes	80.7	3.6	<0.001	37.8	2.5	<0.001	42.9	2.1	<0.001
No	Ref.			Ref.			Ref.		
**Surgery**									
Yes	48.5	3.5	<0.001	34.5	2.4	<0.001	14.0	2.0	<0.001
No	Ref.			Ref.			Ref.		
**Cancer type**									
Leukemia	104.0	4.5	<0.001	67.4	3.1	<0.001	36.6	2.6	<0.001
Brain and CNS cancer	61.4	4.7	<0.001	25.3	3.2	<0.001	36.1	2.7	<0.001
Non-Hodgkin lymphoma	35.9	5.7	<0.001	25.5	3.9	<0.001	10.4	3.4	0.002
Bone & articular cartilage	101.0	6.3	<0.001	83.4	4.4	<0.001	17.6	3.7	<0.001
Mesothelioma Soft tissues	40.0	7.0	<0.001	21.5	4.8	<0.001	18.4	4.1	<0.001
Other cancers	Ref.			Ref.			Ref.		

## Supplementary Materials

The following are available online at https://www.mdpi.com/1660-4601/17/17/6020/s1, Supplementary Table S1. Total medical cost to childhood cancer survivors from initial diagnosis by cancer types (mean ± S.D.); Supplementary Table S2. Result of regression analysis on total medical cost on childhood cancer survivors from the diagnosis by cancer types; Supplementary Table S3. Cumulative medical cost† of each childhood cancer survivors by treatment phase and socioeconomic status.
