# Supplementary material for: The Financial Burden Associated with Medical Costs among Childhood Cancer Patients and Their Families Related to Their Socioeconomic Status: The Perspective of National Health Insurance Service
Source: Int J Environ Res Public Health. 2020 Aug 19;17(17):6020. doi: 10.3390/ijerph17176020 (PMC7503756; doi:10.3390/ijerph17176020)
Supplement: Supplementary file 1 [file ijerph-17-06020-s001.pdf]

Supplementary Table S1. Total medical cost to childhood cancer survivors from initial diagnosis by cancer types (mean ± S.D.)

Variables	Total medical cost†																	
	Leukemia			Brain & Central Nervous System cancer			Non-Hodgkin lymphoma			Bone & Articular cartilage cancer			Mesothelioma Soft tissues			Other cancers		
	**Mean**	**±**	**S.D**	**Mean**	**±**	**S.D**	**Mean**	**±**	**S.D**	**Mean**	**±**	**S.D**	**Mean**	**±**	**S.D**	**Mean**	**±**	**S.D**
	**53,468**		**39,600**	**36,268**	**±**	**37,350**	**37,292**	**±**	**35,574**	**52,049**	**±**	**39,766**	**35,686**	**±**	**36,204**	**21,204**	**±**	**28,663**
**Sex**																		
Male	53,994	±	40,000	37,930	±	38,345	38,142	±	36,343	50,808	±	39,147	38,001	±	36,692	23,742	±	31,778
Female	52,749	±	39,060	33,976	±	35,848	35,298	±	33,707	53,690	±	40,602	33,024	±	35,554	19,057	±	25,549
**Age at diagnosis**																		
0-4	46,615	±	33,798	43,155	±	41,861	33,788	±	38,441	29,765	±	31,442	39,570	±	35,920	27,846	±	32,814
5-9	48,584	±	35,815	39,360	±	41,102	41,684	±	36,976	46,985	±	41,658	42,216	±	42,548	24,551	±	34,203
10-14	63,295	±	44,854	33,645	±	33,692	38,463	±	31,095	56,044	±	40,503	28,228	±	31,668	17,557	±	24,418
15-17	66,014	±	45,942	29,213	±	31,117	33,202	±	37,557	53,939	±	37,451	29,844	±	35,473	13,357	±	18,626
**Household income level**																		
Low	46,166	±	39,349	35,064	±	36,261	34,222	±	33,461	50,988	±	40,925	35,285	±	39,889	19,043	±	26,564
Mid-low	50,966	±	37,281	34,565	±	35,813	35,349	±	37,272	45,427	±	42,759	36,064	±	35,985	21,460	±	28,334
Mid-high	57,734	±	40,841	36,947	±	37,375	38,533	±	36,331	52,717	±	34,746	35,028	±	33,217	23,341	±	31,219
High	66,508	±	38,690	40,548	±	41,739	43,085	±	34,569	61,911	±	37,673	36,947	±	34,930	21,878	±	29,105
**Treated hospital type**																		
Tertiary hospital	53,512	±	39,594	36,448	±	37,405	37,389	±	35,581	52,896	±	39,598	35,961	±	36,222	21,434	±	28,772
Other	8,846	±	10,187	11,439	±	15,621	4,537	±	5,279	4,796	±	7,555	1,862	±	1,882	4,253	±	8,410
**Hospital region**																		
Capital Area	54,860	±	39,589	36,968	±	36,923	39,830	±	35,248	56,352	±	35,017	40,681	±	37,541	24,059	±	31,167
Metropolitan	50,670	±	39,093	37,411	±	41,911	27,662	±	30,718	46,954	±	55,467	28,957	±	28,347	18,657	±	27,043
Rural Area	51,347	±	39,699	32,657	±	35,708	34,951	±	37,312	38,187	±	42,133	22,957	±	31,208	16,022	±	21,931
**Chemotherapy**																		
Yes	56,961	±	38,607	59,234	±	39,152	45,437	±	35,147	67,820	±	33,792	52,400	±	35,686	35,849	±	33,422
No	7,697	±	18,239	15,394	±	18,852	4,891	±	8,503	6,767	±	8,703	7,989	±	11,967	5,919	±	7,487
**Radiotherapy**																		

Yes	78,466	±	42,439	51,700	±	38,682	59,406	±	44,827	63,322	±	39,866	64,529	±	39,522	35,590	±	38,923
No	47,844	±	36,667	20,184	±	28,004	33,243	±	32,039	50,853	±	39,610	23,102	±	26,142	16,112	±	21,853
**Surgery**																		
Yes	98,946	±	42,117	44,346	±	40,294	49,090	±	40,750	60,914	±	37,839	40,095	±	38,034	24,512	±	31,865
No	45,213	±	32,986	19,894	±	23,093	26,372	±	25,590	13,228	±	19,677	18,797	±	20,927	13,693	±	17,347
**Medical service**																		
Inpatient	43,623		35,607	24,509	±	28,872	28,932	±	30,924	43,473	±	34,504	26,003	±	29,334	15,292	±	23,065
Outpatient	9,844		10,863	11,759	±	13,864	8,360	±	7,809	8,575	±	8,305	9,683	±	9,794	5,912	±	8,021

†Presented in KRW (KRW 1,200 = USD 1) ‡Unit: 1,000 KRW

Supplementary Table S2. Result of regression analysis on total medical cost on childhood cancer survivors from the diagnosis by cancer types

	Leukemia			Brain & Central Nervous System cancer			Non-Hodgkin lymphoma		
	**β**	**S.E**	**P-value**	**β**	**S.E**	**P-value**	**β**	**S.E**	**P-value**
**Sex**									
Male	836,872	1,407,483	0.552	94,711	1,746,988	0.957	2,331,431	2,528,811	0.357
Female	Ref.			Ref.			Ref.		
**Age at diagnosis**									
0-4	-14,584,177	2,385,107	<.001	12,450,270	2,884,368	<.001	1,934,374	3,834,783	0.614
5-9	-11,415,211	2,437,314	<.001	7,298,551	2,634,312	0.006	4,644,137	3,319,420	0.162
10-14	-752,637	2,477,536	0.761	1,800,376	2,487,890	0.469	-1,363,358	3,095,207	0.66
15-17	Ref.			Ref.			Ref.		
**Household income level**									
Low	-10,651,896	2,216,395	<.001	-4,437,886	2,686,130	0.099	-4,211,034	3,446,032	0.222
Mid-low	-8,219,093	2,213,367	<.001	-6,325,456	2,679,531	0.018	-3,241,599	3,437,882	0.346
Mid-high	-4,920,631	2,280,399	0.031	-1,400,541	2,745,456	0.610	-2,260,307	3,524,510	0.522
High	Ref.			Ref.			Ref.		
**Treated hospital type**									
Tertiary hospital	-11,178,789	22,249,488	0.615	-7,037,596	10,284,450	0.494	18,627,052	21,227,486	0.381
Other	Ref.			Ref.			Ref.		
**Hospital region**									
Capital Area	-1,624,154	1,575,714	0.303	-4,030,423	2,332,431	0.084	-2,674,067	2,711,456	0.324
Metropolitan Area	-2,222,317	2,684,135	0.408	2,944,945	3,224,021	0.361	-1,597,426	4,309,150	0.711
Rural Area	Ref.			Ref.			Ref.		
**Chemotherapy**									
Yes	41,433,148	2,776,405	<.001	34,613,699	1,987,569	<.001	34,914,124	3,034,258	<.001
No	Ref.			Ref.			Ref.		
**Radiotherapy**									
Yes	17,187,175	1,855,259	<.001	14,316,242	2,015,649	<.001	20,071,633	3,281,623	<.001
No	Ref.			Ref.			Ref.		
**Surgery**									
Yes	45,272,419	1,978,982	<.001	14,871,016	1,875,789	<.001	16,866,343	2,347,348	<.001
No	Ref.			Ref.			Ref.		

	Bone & Articular cartilage cancer			Mesothelioma Soft tissues			Other cancers		
	**β**	**S.E**	**P-value**	**β**	**S.E**	**P-value**	**β**	**S.E**	**P-value**
**Sex**									
Male	40,667	2,514,519	0.987	307,766	2,772,949	0.912	3,299,845	893,813	<.001
Female	Ref.			Ref.			Ref.		
**Age at diagnosis**									
0-4	-11,009,650	5,133,298	0.033	4,465,543	4,239,393	0.293	11,092,453	1,222,607	<.001
5-9	-1,299,568	3,970,167	0.744	4,924,953	5,196,387	0.344	8,085,063	1,509,013	<.001
10-14	5,608,614	2,971,163	0.06	2,047,242	4,499,836	0.649	4,409,422	1,257,460	0.001
15-17	Ref.			Ref.			Ref.		
**Household income level**									
Low	-5,441,040	3,682,138	0.14	-1,080,770	4,645,557	0.816	-2,555,293	1,340,521	0.057
Mid-low	-2,689,298	3,582,205	0.453	-3,684,314	4,522,067	0.416	-235,815	1,371,490	0.864
Mid-high	-1,710,960	3,714,359	0.056	-1,415,410	4,664,025	0.762	-467,680	1,400,694	0.739
High	Ref.			Ref.			Ref.		
**Treated hospital type**									
Tertiary hospital	-11,109,303	9,916,637	0.263	310,689	15,576,622	0.984	-3,164,421	3,874,816	0.414
Other	Ref.			Ref.			Ref.		
**Hospital region**									
Capital Area	332,584	3,473,007	0.924	4,703,840	3,479,670	0.177	3,061,726	1,042,981	0.003
Metropolitan Area	11,997,102	4,698,020	0.011	-2,514,190	5,522,980	0.649	2,064,178	1,467,679	0.16
Rural Area	Ref.			Ref.			Ref.		
**Chemotherapy**									
Yes	53,393,437	3,210,134	<.001	32,662,924	3,224,499	<.001	26,556,557	917,441	<.001
No	Ref.			Ref.			Ref.		
**Radiotherapy**									
Yes	5,208,673	4,253,775	0.221	27,363,034	3,304,990	<.001	18,779,016	1,039,004	<.001
No	Ref.			Ref.			Ref.		
**Surgery**									
Yes	24,512,017	3,668,370	<.001	11,769,426	3,492,065	0.001	8,425,458	980,295	<.001
No	Ref.			Ref.			Ref.		

Supplementary Table S3. Cumulative medical cost of each childhood cancer survivors by treatment phase and socioeconomic status

Household Income	Treatment Phase	Total medical cost†					
		Leukemia	Brain & CNS cancer	Non-Hodgkin lymphoma	Bone & Articular cartilage cancer	Meso-thelioma Soft tissues	Other cancers
**Low**	0-3month	15,712	11,773	13,851	14,786	7,607	6,658
	≤6month	30,012	20,006	25,549	30,409	16,070	11,160
	≤9month	36,745	25,842	30,583	41,892	25,750	14,745
	≤1year	40,858	32,511	34,890	49,004	33,367	17,640
	≤2year	48,777	40,366	41,607	56,654	44,864	21,990
	≤3year	53,943	44,788	44,924	63,577	49,056	24,302
	≤4year	56,733	51,130	47,473	67,173	53,611	25,700
	≤5year	58,774	55,296	48,978	72,217	57,026	27,008
**Mid low**	0-3month	17,300	12,477	13,334	13,134	9,720	6,868
	≤6month	30,325	20,647	24,284	28,345	17,558	12,275
	≤9month	37,218	26,281	29,284	41,668	25,581	15,620
	≤1year	42,448	32,217	33,616	48,102	32,319	19,009
	≤2year	50,214	39,136	39,430	55,292	42,490	24,032
	≤3year	55,523	43,626	44,877	63,002	45,550	26,516
	≤4year	58,802	48,766	47,955	67,765	49,668	28,850
	≤5year	61,317	53,870	49,487	71,883	51,889	29,851
**Mid high**	0-3month	19,617	12,168	14,556	16,727	9,253	7,474
	≤6month	33,200	20,551	23,510	30,675	17,565	12,785
	≤9month	41,011	26,495	27,397	40,605	25,496	17,083
	≤1year	46,112	31,900	31,439	46,525	32,372	20,978
	≤2year	53,891	39,633	38,040	53,651	39,692	26,379
	≤3year	58,579	45,228	42,532	58,421	44,645	28,831
	≤4year	62,073	49,722	44,348	62,124	47,357	30,554
	≤5year	64,042	54,809	47,462	65,395	48,636	32,266
**High**	0-3month	22,987	13,789	16,739	19,218	9,995	7,332
	≤6month	37,247	21,665	25,874	34,296	19,102	12,266
	≤9month	44,787	28,402	31,591	45,792	26,337	16,345
	≤1year	49,932	35,914	34,521	50,319	33,454	20,609
	≤2year	58,833	45,930	39,859	58,094	41,538	25,381
	≤3year	63,917	51,950	43,034	64,079	44,913	27,766
	≤4year	66,259	55,796	45,745	65,485	46,554	29,165
	≤5year	67,927	59,358	47,088	68,287	49,614	30,405

†Presented in KRW (KRW 1,200 = USD 1)   ‡Unit: 1,000 KRW
